# Differential expression of Cdk5-phosphorylated CRMP2 following a spared nerve injury

**DOI:** 10.1186/s13041-020-00633-1

**Published:** 2020-06-22

**Authors:** Aubin Moutal, Yingshi Ji, Shreya Sai Bellampalli, Rajesh Khanna

**Affiliations:** 1 Departments of Pharmacology, University of Arizona, Tucson, AZ 85724 USA; 2 Department of Pharmacology, College of Basic Medical Sciences, Jilin University, Changchun, Jilin, 130021 People’s Republic of China; 3 Mayo Clinic School of Medicine, 26 Mayo Park Dr SE, Rochester, MN 55904 USA; 4 Departments of Anesthesiology, University of Arizona, Tucson, AZ 85724 USA; 5 Neuroscience Graduate Interdisciplinary Program, College of Medicine, University of Arizona, Tucson, AZ 85724 USA; 6 BIO5 Institute, 657 East Helen Street, P.O. Box 210240, Tucson, AZ 85724 USA; 7 The Center for Innovation in Brain Sciences, The University of Arizona Health Sciences, Tucson, AZ 85724 USA

**Keywords:** Cdk5, CRMP2, Neuropathic pain, DRGs, CaV2,2, NaV1.7

## Abstract

Effective treatment of high-impact pain patients is one of the major stated goals of the National Pain Strategy in the United States. Identification of new targets and mechanisms underlying neuropathic pain will be critical in developing new target-specific medications for better neuropathic pain management. We recently discovered that peripheral nerve injury-induced upregulation of an axonal guidance phosphoprotein collapsin response mediator protein 2 (CRMP2) and the N-type voltage-gated calcium (CaV2.2) as well as the NaV1.7 voltage-gated sodium channel, correlates with the development of neuropathic pain. In our previous studies, we found that interfering with the phosphorylation status of CRMP2 is sufficient to confer protection from chronic pain. Here we examined the expression of CRMP2 and CRMP2 phosphorylated by cyclin-dependent kinase 5 (Cdk5, on serine residue 522 (S522)) in sciatic nerve, nerve terminals of the glabrous skin, and in select subpopulations of DRG neurons in the SNI model of neuropathic pain. By enhancing our understanding of the phosphoregulatory status of CRMP2 within DRG subpopulations, we may be in a better position to design novel pharmacological interventions for chronic pain.

## Introduction

Chronic neuropathic pain can be characterized by allodynia (painful response to a non-painful stimulus), hyperalgesia (increase sensitivity to pain) or spontaneous pain. These symptoms may be alleviated by pharmacological inactivation of the damaged nerve [1, 2]. Thus, modulating the signal emanating from the dorsal root ganglia (DRG) sensory neurons is key to mitigating neuropathic pain sensations. In clinically defined genetic pain syndromes where patients experience uncontrollable pain during their lifetimes, activating mutations of voltage-gated sodium channels (NaV1.7, NaV1.8 and NaV1.9), restricted to the DRGs, have been reported [3]. Together, these findings highlight that dysregulation of DRG function may be sufficient for expression of chronic pain.

Our laboratory has been investigating proteins involved in dysregulation of nociceptive signaling, specifically within the periphery (i.e. in the DRG) that may lead to chronic pain sensations. Inspired by the idiopathic pain reported by patients with Neurofibromatosis type 1 (NF1), we found that deleting the *Nf1* gene (coding for the protein neurofibromin) using clustered regularly interspaced short palindromic repeats and CRISPR-associated (Cas9) (CRISPR/Cas9) in DRG could elicit hyperalgesic behaviors [4–6]. Following *Nf1* gene editing, we detected increased function of two key voltage-gated ion channels implicated in pain – the N-type or CaV2.2 voltage-gated calcium channel and the NaV1.7 voltage-gated sodium channel – in DRG neurons [4]. A function of neurofibromin is to bind to the cytosolic collapsin response mediator protein 2 (CRMP2) to inhibit its phosphorylation [7]. Unphosphorylated CRMP2 functions as an axonal growth protein [8]. Upon phosphorylation by cyclin-dependent kinase 5 (Cdk5, on serine residue 522 (S522)), CRMP2 binds to CaV2.2 and NaV1.7 to maintain their membrane localization and function [8]. Loss of the negative regulation of neurofibromin over CRMP2 allowed for increased CRMP2 phosphorylation by Cdk5 [4, 7, 9], consequently upregulating both CaV2.2 and NaV1.7 [8] and culminating in a decrease of threshold for thermal pain sensations.

Additionally, we reported that CRMP2 phosphorylation by Cdk5 was increased in both DRG and spinal dorsal horn in a rat neuropathic pain model (spared nerve injury (SNI)) [10]. Phosphorylated (S522) CRMP2 could not only control the pre-synaptic localization of CaV2.2 and NaV1.7 but also affected spontaneous excitatory post-synaptic currents in the spinal dorsal horn [11]. Targeting CRMP2 phosphorylation either genetically (by expressing a S522A mutant that is resistant to phosphorylation) [10] or pharmacologically (using the CRMP2 phosphorylation inhibitor (*S*)-N-benzyl 2-acetamido-3-methoxypropionamide ((*S*)-Lacosamide)) [12], reversed the allodynic behaviors evoked by a mechanical stimulus. Conversely, expressing a CRMP2 mutant (S522D, mimicking constitutive phosphorylation) induced mechanical allodynia [10]. These observations led us to conclude that CRMP2 phosphorylation by Cdk5 was a key pathological dysregulation occurring in DRG neurons and underlying chronic neuropathic pain [8].

While our past work provided key insights into how Cdk5 controls CRMP2 phosphorylation to contribute to pain signaling, we have not previously explored the exact population(s) of DRGs wherein Cdk5 phosphorylation predominates. Therefore, here we aimed to study the populations of DRGs that express both CRMP2 and phosphorylated CRMP2 under naïve conditions as well conditions of a neuropathic pain model; the SNI model was chosen because we previously reported in this model of chronic neuropathic pain that CRMP2 phosphorylation by Cdk5 was increased in DRGs and in spinal cord [8]. Additionally, we aimed to determine whether CRMP2 phosphorylation was increased in select subpopulations of DRG neurons in the SNI model of neuropathic pain.

## Results

### Development of allodynia after spared nerve injury

**Fig. 1:**
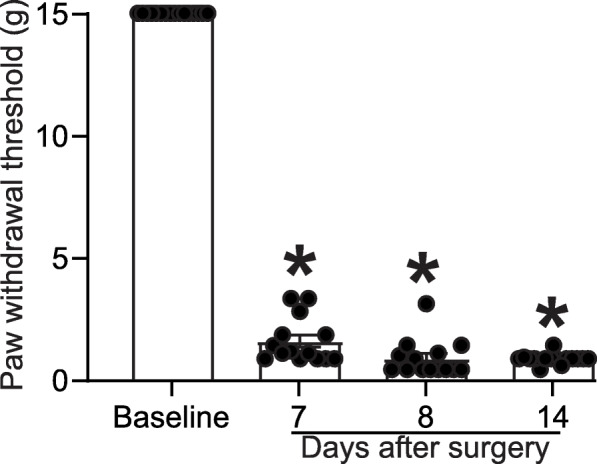
Spared nerve injury induces stable mechanical allodynia. Male rats developed allodynia 7 days after surgery. Mechanical allodynia was measured by the Von Frey method. Paw withdrawal thresholds were at 15 g before surgery and significantly lowered 7 days after SNI, indicating an increased sensitivity to a non-noxious touch stimulus. Bar graph with scatter plot at the indicated time points (*n* = 14; *p < 0.05 compared to pre-surgery baseline, Kruskal-Wallis test)

In the spared nerve injury model (SNI) of chronic neuropathic pain, we previously demonstrated using whole tissue western blots, that CRMP2 phosphorylation by Cdk5 was increased in DRGs and in spinal cord [10]. This post-translational modification could control the pre-synaptic localization of CaV2.2 and NaV1.7 as well as the ensuing mechanical allodynia. Following surgery where the peroneal and tibial nerves are tightly ligated and axotomized, animals develop mechanical allodynia only on the ipsilateral side of the injury (Fig. 1). To study the localization of the phosphorylated CRMP2 following this neuropathic pain injury, we collected tissues at 14 days after surgery. The contralateral side from the same animal serves as a control because it represents the same animal and micro-environmental conditions but does not express mechanical allodynia.

### Phosphorylated CRMP2 is localized in nerve projections in the sciatic nerve

**Fig. 2:**
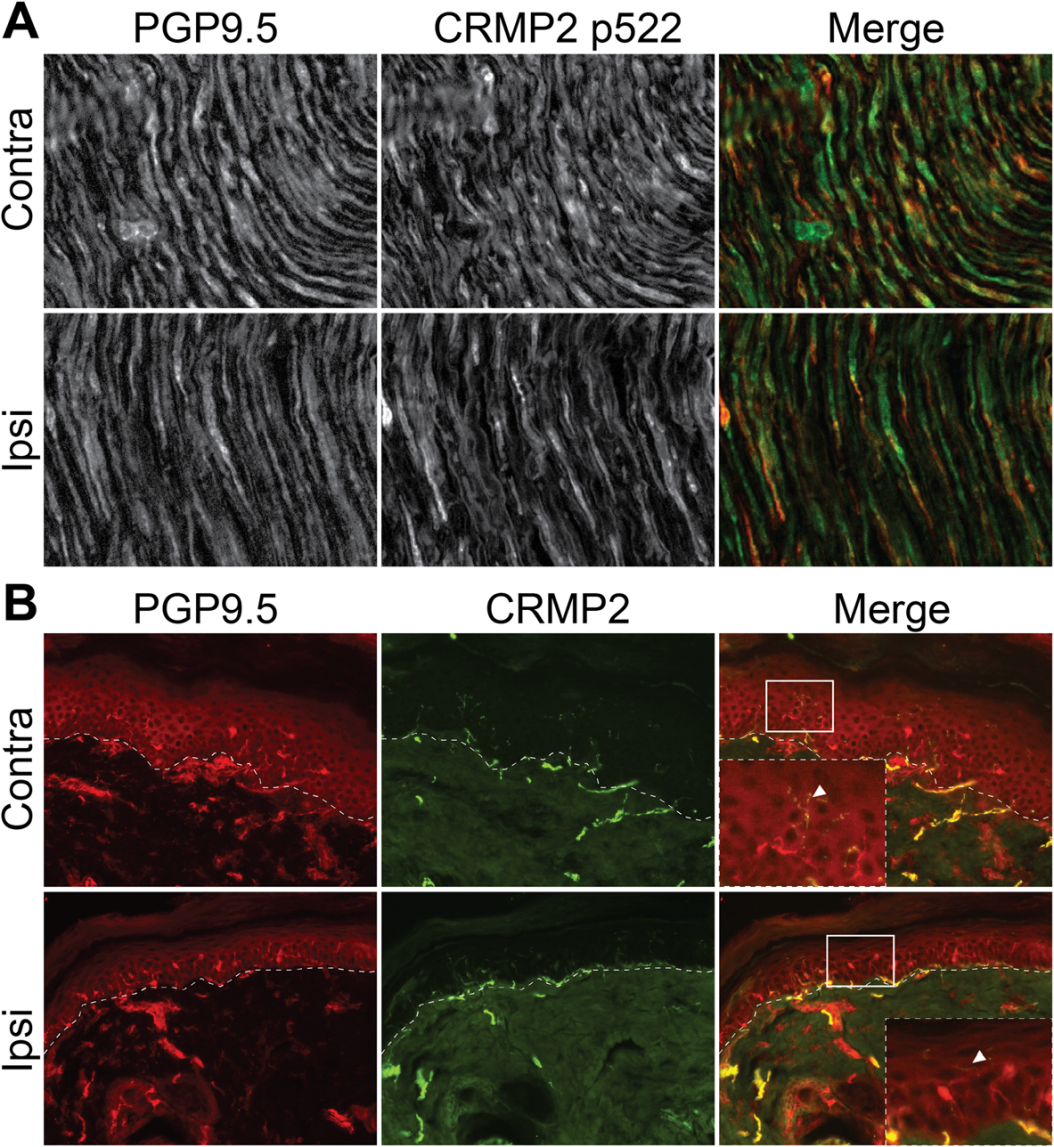
CRMP2 is associated with nerve projections in the sciatic nerve and in the glabrous skin. **a** Micrographs of a 10 μm section of adult sciatic nerve from animals having received a spared nerve injury and immunostained with antibodies against PGP9.5 (to stain nerve fibers) or CRMP2 p522 as indicated. **b** Micrographs of a 10 μm section of adult glabrous skin from animals having received a spared nerve injury and immunostained with antibody against CRMP2 and PGP9.5 to stain nerve fibers skin. The dashed line represents the division between the epidermis and dermis. CRMP2 immunoreactivity was found in the nerve fibers innervating the skin. N = 4 animals. Unless otherwise stated, ipsi = ipsilateral (injured side) and contra = contralateral (non-injured side)

To date, the potential involvement of CRMP2 in pain signal transmission has mostly been studied in the DRG or in the spinal cord [4, 13–15]. The pattern of CRMP2 expression and phosphorylation in the sciatic nerve is unknown. Thus, we performed immunostainings for CRMP2 p522 in the sciatic nerve (Fig. 2a). We found CRMP2 p522 expression in the nerves, co-stained with the marker protein gene product 9.5 (PGP9.5)/ubiquitin-C-terminal hydrolase 1 (UCHL-1), but not with the myelin sheath. We also could not observe any CRMP2 p522 staining around the nodes of Ranvier. CRMP2 p522 staining intensity was unchanged between the contralateral and the ipsilateral sides. This shows that CRMP2 might travel from the DRG through the nerve projections to reach distal sites.

### CRMP2 is localized in nerve endings of the glabrous skin

The glabrous skin is rich in nerve terminals that are essential for sensing a mechanical stimulus. These fibers can be sensitized in neuropathic pain states. Thus, we stained for CRMP2 in the glabrous skin collected from both the ipsilateral (i.e., the injured side) and the contralateral side of SNI rats. CRMP2 staining revealed a specific pattern corresponding to the nerve terminals in the glabrous skin (Fig. 2b). The staining pattern and intensity was unchanged between SNI and control sides. Staining for CRMP2 p522 did not show any specific signal in the glabrous skin and thus could not be used. These results show, for the first time, CRMP2 expression in nerve terminals of the glabrous skin.

### CRMP2 expression and phosphorylation are dysregulated in specific L4 DRG neuronal populations

**Fig. 3:**
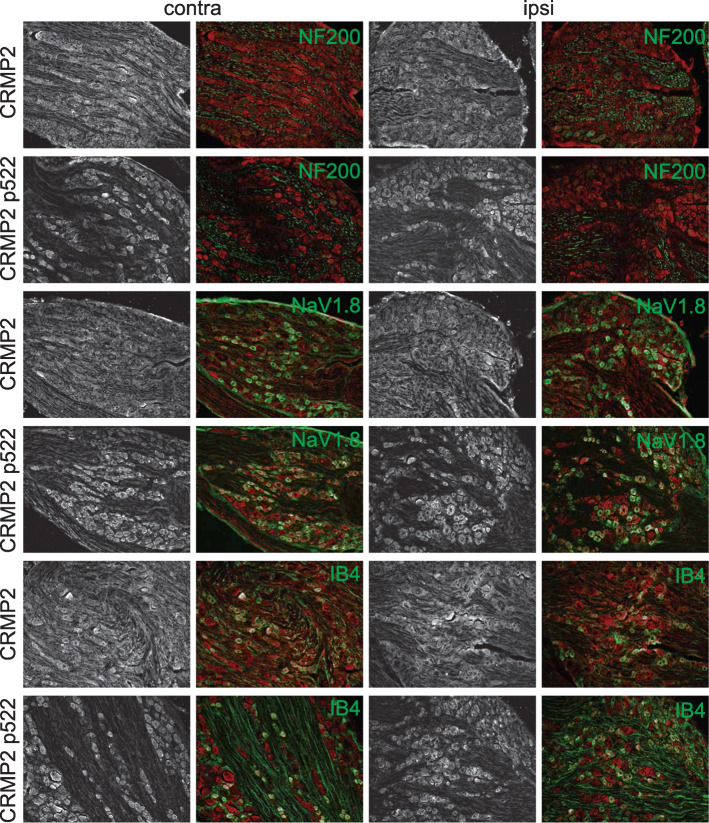
CRMP2 expression and phosphorylation is detected in L4 DRG neuron subpopulations. Micrographs of a 10 μm section of adult L4 DRG from animals having received a spared nerve injury and immunostained with antibodies against CRMP2, CRMP2 p522, NF200, NaV1.8 or for the lectin IB4 as indicated. Micrographs are representative of *N* = 4 animals with 8 independent slices per animal per staining combination; ipsi = ipsilateral (injured side) and contra = contralateral (non-injured side)

**Fig. 4:**
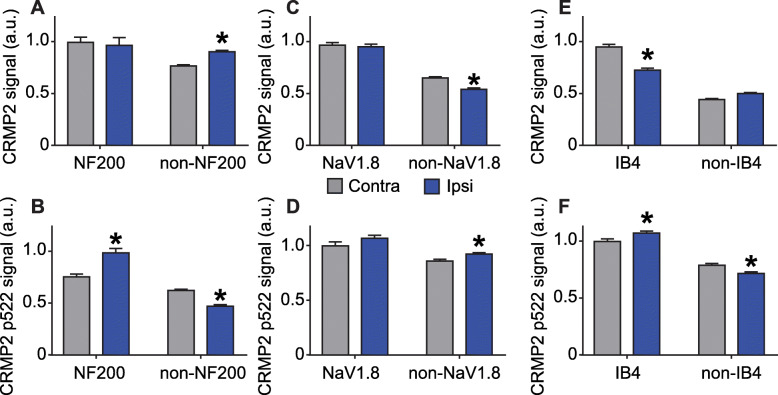
Alterations of CRMP2 expression and phosphorylation levels in L4 DRG neuron subpopulations. Bar graphs showing the comparison between contralateral and ipsilateral side of a spared nerve injury of total CRMP2 or CRMP2 p522 signal depending of NF200, NaV1.8 of IB4 neuronal subpopulations. **a** total CRMP2 level in NF200 neurons (contra, *n* = 1870 individual neurons, ipsi *n* = 1514 individual neurons). **b** CRMP2 p522 level in NF200 neurons (contra, *n* = 1044 individual neurons, ipsi *n* = 882 individual neurons). **c** total CRMP2 level in NaV1.8 neurons (contra, *n* = 1237 individual neurons, ipsi *n* = 934 individual neurons). **d** CRMP2 p522 level in NaV1.8 neurons (contra, *n* = 860 individual neurons, ipsi *n* = 1088 individual neurons). **e** total CRMP2 level in IB4 neurons (contra, *n* = 1153 individual neurons, ipsi n = 1183 individual neurons). **f** CRMP2 p522 level in IB4 neurons (contra, *n* = 950 individual neurons, ipsi *n* = 1004 individual neurons). *p < 0.05; Student’s t-test compared to the contralateral side

Cdk5-phosphorylated CRMP2 levels were increased in ipsilateral DRGs in SNI rats [10]. These western blot analyses were done by combining L4, L5 and L6 DRGs because of their involvement in this pain model. Consequently, this result is an average of all possible changes in each DRG after SNI. This model involves a transection and ligation of the common peroneal and tibial nerves, while the sural nerve is left intact [16]. To more precisely identify possible differences in CRMP2 phosphorylation levels, L4, L5 or L6 DRGs were separately isolated from either the contralateral or the ipsilateral sides, and then CRMP2 and CRMP2 p522 levels in DRG neurons were explored by co-staining with markers of specific DRG subpopulations. We focused this study on neurofilament 200 (NF200) positive neurons (a marker for A-fiber neurons), NaV1.8 positive neurons (marker for nociceptors), and isolectin B4 (IB4) positive neurons (marker for non-peptidergic neurons). L4 DRG was co-stained for these markers and either CRMP2 or CRMP2 p522 (Fig. 3). We found that CRMP2 expression is increased in non-NF200 neurons (Fig. 4a, Table 1) while CRMP2 p522 levels were increased in NF200^+^ neurons and decreased in non-NF200 neurons (Fig. 4b, Table 1) on the ipsilateral side compared to the contralateral side of L4 DRG. Decreased CRMP2 (Fig. 4c, Table 1) but increased CRMP2 p522 levels (Fig. 4d, Table 1) were detected in non-NaV1.8 neurons on the ipsilateral side of L4 DRG. In IB4^+^ neurons of the L4 DRG, we observed decreased CRMP2 (Fig. 4e, Table 1) and increased CRMP2 p522 (Fig. 4f, Table 1) signal, while in IB4^−^ neurons, CRMP2 level was unchanged and CRMP2 p522 was decreased. In summary, increased Cdk5-phosphorylated CRMP2 levels in L4 DRG was restricted to sensory neurons from the NF200^+^, NaV1.8^−^ and IB4^+^ populations.

**Table 1:** Summary table of normalized fluorescence values for the indicated staining across DRGs L4–6

		CRMP2					pS522-CRMP2				
		contra		Ipsi			contra		Ipsi		
DRG	Marker	Mean	SD	Mean	SD	*p*-value	Mean	SD	Mean	SD	*p*-value
L4	NF200+	1.000	0.515	0.971	0.634	0.794	1.000	0.499	1.308	0.360	**0.001**
	NF200-	0.772	0.560	0.909	0.589	0.090	0.827	0.396	0.625	0.868	0.268
	Nav1.8+	1.000	0.391	0.983	0.344	0.804	1.000	0.366	1.070	0.368	0.305
	NaV1.8-	0.672	0.503	0.559	0.291	0.115	0.861	0.437	0.926	0.373	0.359
	IB4+	1.000	0.354	0.763	0.242	**0.002**	1.000	0.417	1.073	0.322	0.290
	IB4-	0.465	0.752	0.527	0.319	0.276	0.790	0.403	0.718	0.498	0.330
L5	NF200+	1.000	0.377	0.975	0.251	0.678	1.000	0.420	1.366	0.325	**0.000**
	NF200-	0.840	0.395	0.912	0.312	0.196	0.914	0.494	1.110	0.369	**0.017**
	Nav1.8+	1.000	0.389	0.812	0.306	**0.023**	1.000	0.331	1.117	0.232	**0.033**
	NaV1.8-	0.775	0.436	0.790	0.386	0.824	0.863	0.313	0.991	0.299	**0.020**
	IB4+	1.000	0.407	1.214	0.614	**0.008**	1.000	0.450	1.188	0.426	**0.026**
	IB4-	0.738	0.517	0.690	0.400	0.537	0.917	0.472	1.170	0.527	**0.007**
L6	NF200+	1.000	0.269	1.002	0.220	0.970	1.000	0.472	1.280	0.284	**0.001**
	NF200-	0.973	0.296	0.847	0.252	**0.036**	0.748	0.436	1.010	0.441	**0.001**
	Nav1.8+	1.000	0.324	1.018	0.280	0.740	1.000	0.526	1.223	0.414	**0.055**
	NaV1.8-	0.926	0.451	0.909	0.326	0.803	1.117	0.600	1.185	0.434	0.502
	IB4+	1.000	0.228	0.951	0.201	0.244	1.000	0.372	1.078	0.554	0.373
	IB4-	0.813	0.349	0.769	0.245	0.399	0.778	0.410	1.232	0.454	**0.000**

Values were normalized to the contralateral staining for each antibody as indicated (n = 15 fields from at least 4 different rats). Significant p-values are indicated in bold font (p < 0.05, t-test compared to the contralateral side). Abbreviations: NaV1.8, voltage-gated sodium channel subunit 1.8; IB4, isolectin B4; DRG, dorsal root ganglia; CRMP2 collapsin response mediator protein; NF200, neurofilament 200; contra, contralateral non injured side; ipsi, ipsilateral injured side

### CRMP2 p522 is increased in all L5 DRG neuronal populations

**Fig. 5:**
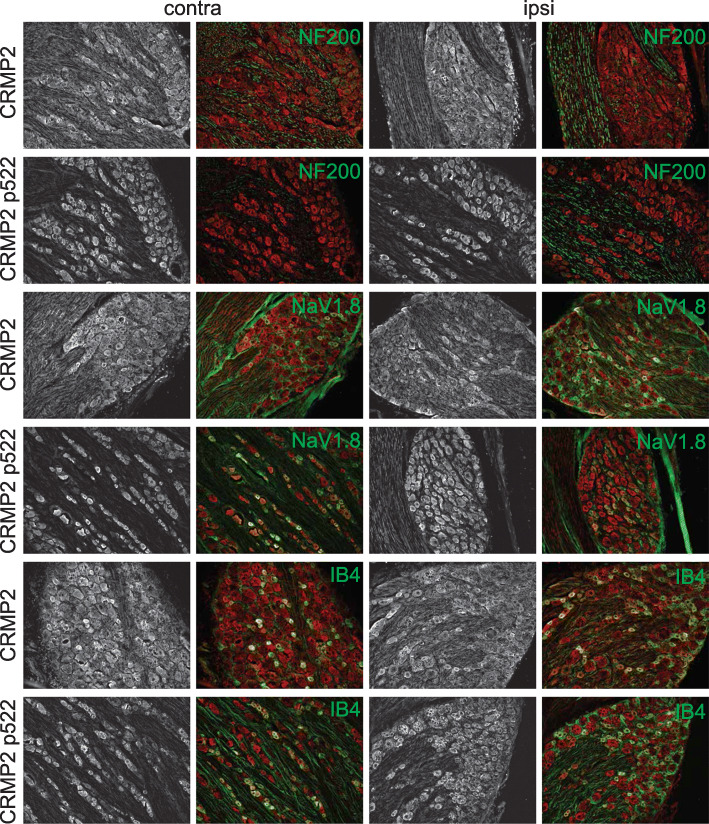
CRMP2 expression and phosphorylation is detected in L5 DRG neuron subpopulations. Micrographs of a 10 μm section of adult L5 DRG from animals having received a spared nerve injury and immunostained with antibodies against CRMP2, CRMP2 p522, NF200, NaV1.8 or for the lectin IB4 as indicated. Micrographs are representative of N = 4 animals with 8 independent slices per animal per staining combination; ipsi = ipsilateral (injured side) and contra = contralateral (non-injured side)

**Fig. 6:**
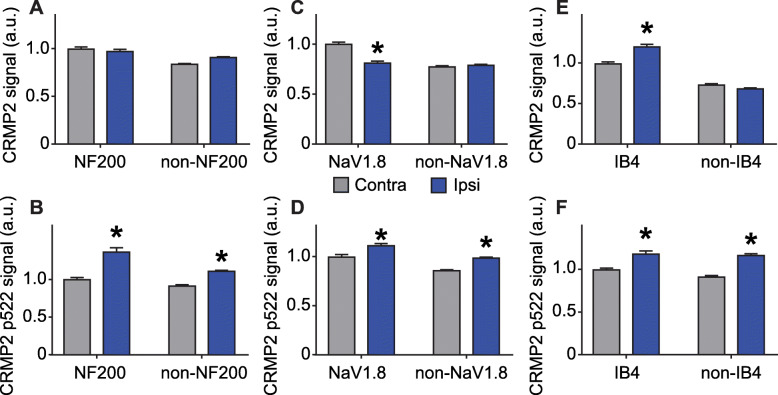
Alterations of CRMP2 expression and phosphorylation levels in L5 DRG neuron subpopulations. Bar graphs showing the comparison between contralateral and ipsilateral side of a spared nerve injury of total CRMP2 or CRMP2 p522 signal depending of NF200, NaV1.8 of IB4 neuronal subpopulations. **a** total CRMP2 level in NF200 neurons (contra, *n* = 1257 individual neurons, ipsi *n* = 1187 individual neurons). **b** CRMP2 p522 level in NF200 neurons (contra, *n* = 1040 individual neurons, ipsi *n* = 899 individual neurons). **c** total CRMP2 level in NaV1.8 neurons (contra, *n* = 1369 individual neurons, ipsi *n* = 1528 individual neurons). **d** CRMP2 p522 level in NaV1.8 neurons (contra, *n* = 1408 individual neurons, ipsi *n* = 885 individual neurons). **e** total CRMP2 level in IB4 neurons (contra, *n* = 772 individual neurons, ipsi *n* = 1251 individual neurons). **f** CRMP2 p522 level in IB4 neurons (contra, *n* = 902 individual neurons, ipsi *n* = 906 individual neurons). *p < 0.05; Student’s t-test compared to the contralateral side

We next asked if we could detect changes in CRMP2 expression and phosphorylation levels within sub-population specific sensory neurons from L5 DRG. The nerve projecting from this DRG is transected in SNI [16]. We stained L5 DRG for CRMP2 or CRMP2 p522 in combination with NF200, NaV1.8 or IB4 (Fig. 5). CRMP2 expression was unchanged between NF200^+^ and NF200^−^ neurons (Fig. 6a, Table 1) for either side. CRMP2 p522 was increased on the SNI side for both NF200^+^ and NF200^−^ neurons (Fig. 6b, Table 1). CRMP2 expression level was decreased in NaV1.8^+^ neurons (Fig. 6c, Table 1) while CRMP2 p522 was increased in both NaV1.8^+^ and NaV1.8^−^ neurons (Fig. 6d, Table 1). Finally, CRMP2 expression level was increased in IB4^+^ neurons but not in IB4^−^ neurons (Fig. 6e, Table 1) and CRMP2 p522 was increased in all L5 sensory neurons, irrespective of their IB4 status (Fig. 6f, Table 1). In summary, SNI resulted in decreased CRMP2 expression in NaV1.8^+^ neurons and increased CRMP2 expression in IB4^+^ neurons. CRMP2 phosphorylation by Cdk5 was enhanced in all neurons in L5 DRG, independent of their NF200, NaV1.8 or IB4 status.

### CRMP2 p522 is increased in non-IB4 L6 DRG neurons

**Fig. 7:**
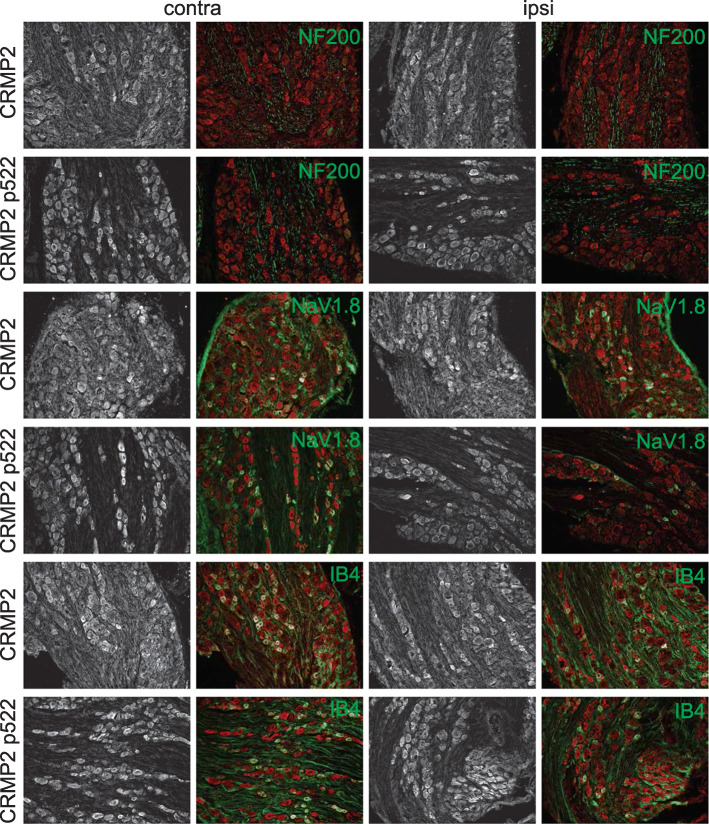
CRMP2 expression and phosphorylation is detected in L6 DRG neuron subpopulations. Micrographs of a 10 μm section of adult L6 DRG from animals having received a spared nerve injury and immunostained with antibodies against CRMP2, CRMP2 p522, NF200, NaV1.8 or for the lectin IB4 as indicated. Micrographs are representative of N = 4 animals with 8 independent slices per animal per staining combination; ipsi = ipsilateral (injured side) and contra = contralateral (non-injured side)

**Fig. 8:**
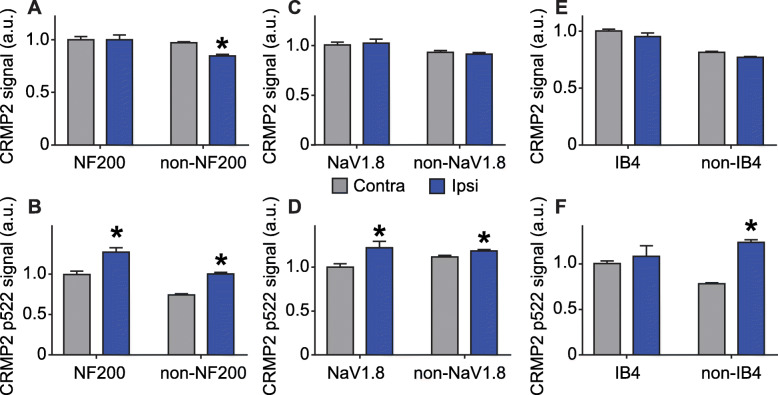
Alterations of CRMP2 expression and phosphorylation levels in L6 DRG neuron subpopulations. Bar graphs showing the comparison between contralateral and ipsilateral side of a spared nerve injury of total CRMP2 or CRMP2 p522 signal depending of NF200, NaV1.8 of IB4 neuronal subpopulations. **a** total CRMP2 level in NF200 neurons (contra, *n* = 949 individual neurons, ipsi *n* = 719 individual neurons). **b** CRMP2 p522 level in NF200 neurons (contra, *n* = 1099 individual neurons, ipsi *n* = 855 individual neurons). **c** total CRMP2 level in NaV1.8 neurons (contra, *n* = 954 individual neurons, ipsi *n* = 919 individual neurons). **d** CRMP2 p522 level in NaV1.8 neurons (contra, *n* = 1417 individual neurons, ipsi *n* = 911 individual neurons). **e** total CRMP2 level in IB4 neurons (contra, *n* = 1118 individual neurons, ipsi *n* = 1189 individual neurons). **f** CRMP2 p522 level in IB4 neurons (contra, *n* = 1236 individual neurons, ipsi *n* = 832 individual neurons). *p < 0.05; Student’s t-test compared to the contralateral side

After discovering the increased CRMP2 p522 levels in all the sensory neurons from L5 DRG, we were interested in characterizing CRMP2 p522 levels in L6 DRG (Fig. 7). The nerve projecting from this DRG is also transected in the SNI model [16]. We found decreased CRMP2 expression in the NF200^−^ neurons on the ipsilateral side of the L6 DRG (Fig. 8a, Table 1). CRMP2 phosphorylation levels were increased in both NF200^+^ and NF200^−^ neurons in the L6 DRG (Fig. 8b, Table 1). No change of CRMP2 expression level was detected in NaV1.8^+^ or NaV1.8^−^ sensory neurons (Fig. 8c, Table 1) but CRMP2 p522 level was increased on the ipsilateral side in both NaV1.8^+^ and NaV1.8^−^ neurons of the L6 DRG (Fig. 8d, Table 1). Finally, CRMP2 expression level was unchanged between contralateral and ipsilateral sides in either IB4^+^ or IB4^−^ neurons of L6 DRG (Fig. 8e, Table 1). Here, CRMP2 p522 level was increased in only the IB4^−^ L6 DRG subpopulation but not in IB4^+^ neurons (Fig. 8f, Table 1). These results identify an increased CRMP2 phosphorylation by Cdk5 in all IB4^−^ neurons of the L6 DRG, irrespective of their NF200 or NaV1.8 status.

### CRMP2 expression and phosphorylation in the dorsal horn of the spinal cord

**Fig. 9:**
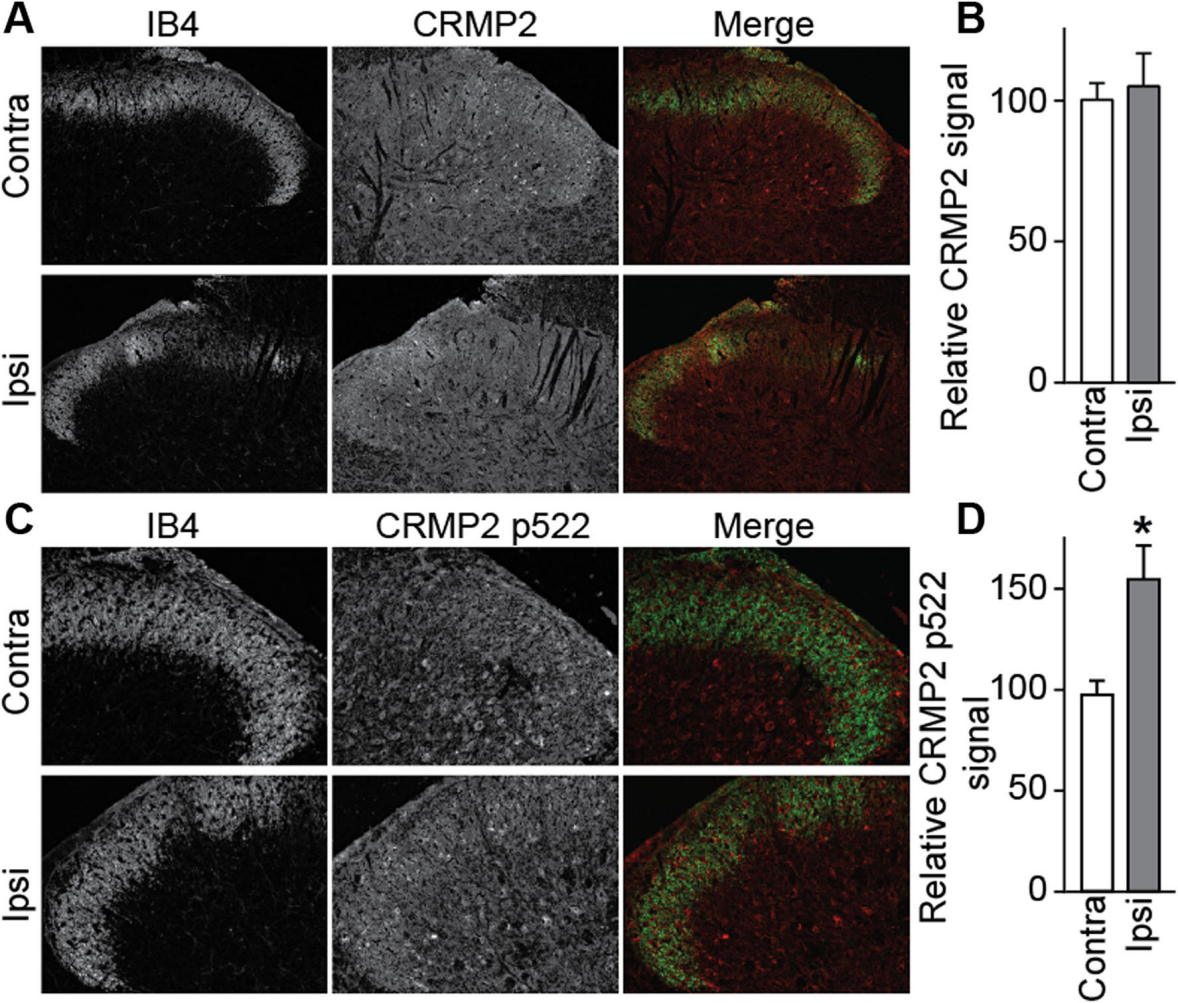
CRMP2 is expressed and phosphorylated in the dorsal horn of the spinal cord. Micrographs of a 20 μm section of adult spinal cord from animals having received a spared nerve injury and immunostained with antibodies against (**a**) total CRMP2 or (**c**) CRMP2 p522 as indicated. IB4 was used to stain superficial laminae of the dorsal horn. CRMP2 and CRMP2 p522 immunoreactivity was found in spinal cord neurons and colocalized with IB4 (indicated by arrows). *N* = 4 animals; ipsi = ipsilateral (injured side) and contra = contralateral (non-injured side). Bar graphs showing the comparison between contralateral and ipsilateral side of a spared nerve injury for (**b**) total CRMP2 or (**d**) CRMP2 p522 signal in the dorsal horn of the spinal cord. Data is shown as mean ± SEM, *p < 0.05; Student’s t-test compared to the contralateral side

We next asked if CRMP2 localization is altered in the dorsal horn of the spinal cord after SNI. We first stained for CRMP2 in the dorsal horn of the spinal cord and found CRMP2 expression in spinal cord neurons (co-expressed with the neuronal soma marker Neurotrace; Supplementary Fig. 1) and in the nerve endings coming from the DRGs (Fig. 9a) in laminae I/II of the dorsal (identified by IB4^+^ staining). No difference of CRMP2 expression level was found on the ipsilateral side compared to the contralateral side of the dorsal horn of the spinal cord (Fig. 9b) similarly to [10]. Next, we examined CRMP2 p522 distribution in the dorsal horn of the spinal cord. We found CRMP2 p522 in spinal cord neurons and identified a staining pattern corresponding to the laminae I/II of the dorsal horn (identified by IB4^+^ staining) (Fig. 9c). On the ipsilateral side, CRMP2 p522 was increased in the dorsal horn of the spinal cord (Fig. 9d). This is consistent with our previous findings [10, 11]. This suggests that, in SNI, CRMP2 p522 is increased in the dorsal horn of the spinal cord.

## Discussion

We report here an immunohistochemical analysis of CRMP2 expression and phosphorylation in sciatic nerve, skin, DRG and spinal cord tissues from rats with a spared nerve injury, a model of chronic neuropathic pain. These results complement our previous western blots analyses [10] and help to better understand the DRG populations that may be involved in CRMP2-related allodynia in neuropathic pain. We found that Cdk5 phosphorylated CRMP2 was increased in most L5/L6 DRG neurons and in a more restricted subpopulation of L4 DRG neurons in SNI. We also identified CRMP2 expression and phosphorylation in the sciatic nerve, the skin nerve terminals and in the dorsal horn of the spinal cord in neuropathic pain.

Cdk5 is a serine/threonine kinase regulating pain signaling [17, 18]. In neuropathic pain, Cdk5 gene expression is epigenetically upregulated [19]. Cdk5 has several substrates in the pain pathway such as CaV3.2 (T-type Ca^2+^ channel) [20], transient receptor potential vanilloid 1 (TRPV1) [21], ATP-gated ion channel (P2X3) [22] and the N-methyl-D-aspartate (NMDA) receptor [23, 24]. Cdk5 substrates are thus directly involved in spinal nociceptive transmission. We have focused here on an intracellular target of cdk5, CRMP2, because this protein can also regulate two other ion channels involved in pain neurotransmission, CaV2.2 and NaV1.7. Another reason for focusing on this kinase comes from a proteomic study which showed that CRMP2 was the main target of the pharmaceutical agent lithium chloride, whose use led to inhibition of CRMP2 phosphorylation by both Cdk5 and glycogen synthase kinase-3β (GSK3β) [25].

Although the interplay between CRMP2 phosphorylation by Cdk5 and the function of CaV2.2 and NaV1.7 has been reported by us, we do not know whether this regulation could be restricted to a subpopulation of DRG neurons. By data mining of single cell RNA-seq data [26], we examined the co-expression of these transcripts in different classes of DRG neurons (Supplementary Fig. 2). Consistent with the data presented in this manuscript, we noted that CRMP2 (*dpysl2*) was expressed in all classes of DRG neurons. Similarly, Cdk5, NaV1.7 (*Scn9a*) and CaV2.2 (*Cacna1b*) could be found in all DRG classes. This suggests that in all DRG neurons, CRMP2 can be phosphorylated by Cdk5 to regulate both of these ion channels. We also analyzed the expression pattern of the protein inhibiting CRMP2 phosphorylation by Cdk5, Neurofibromin. Neurofibromin (*Nf1*) expression level was low but could be found in all DRG classes. In the non-peptidergic DRG neuron subpopulation (i.e., IB4 positive), NaV1.7 expression was higher compared to other subclasses. All these DRG neurons are positive for NaV1.8 (nociceptors). In these neurons we found that CRMP2 expression was decreased in the L4 DRG and increased in the L5 DRG while CRMP2 phosphorylation level was increased in IB4 positive DRG neurons only in the L5 DRG (where all subclasses showed an increased CRMP2 phosphorylation level). This suggests that the increased CRMP2 phosphorylation that we described in SNI, may not be related to increased function of the non-peptidergic DRG neurons in L4 and L6 DRGs but rather restricted to increased activity of the L5 nociceptors. Other classes of DRG neurons that are positive for NaV1.8 are the PEP1–4 (Supplementary Fig. 2). These neurons also express TRPV1 and calcitonin gene related peptide (CGRP). CRMP2 expression and phosphorylation by Cdk5 positively regulates CGRP release from primary afferents [5, 6, 10, 11]. Thus, the increased phosphorylation of CRMP2 in L5 and L6 nociceptors may facilitate the release of the nociceptive neurotransmitter CGRP from these neurons. It is worth noting that neurofibromin expression was higher in the neurofilament classes of DRG neurons where CaV2.2 and NaV1.7 expression is lower compared to other classes. These neurons are part of the myelinated fibers (NF200 positive), where we found CRMP2 phosphorylation by Cdk5 to be increased in all DRG in neuropathic pain conditions. This may indicate that in chronic neuropathic pain, neurofibromin expression could be decreased in the NF200 neurons which would in turn allow for increased CRMP2 phosphorylation.

We have studied the role of CRMP2 expression and phosphorylation in chronic neuropathic pain and found that this single post-translational modification was necessary and sufficient for mechanical allodynia [4, 11, 12, 27, 28]. While we know that this phosphorylation is mediated by Cdk5, understanding the causes of Cdk5 activation leading to increased CRMP2 phosphorylation in the DRG will be important to designing novel treatments to reverse chronic neuropathic pain. To inhibit CRMP2 phosphorylation by Cdk5, we identified (*S*)-Lacosamide [29], which bound to CRMP2 [29], inhibited its phosphorylation [11, 29] and regulated the pre-synaptic localization of CaV2.2 and NaV1.7 [11]. Consequently, (*S*)-Lacosamide reversed mechanical allodynia and thermal hyperalgesia in several models of chronic neuropathic pain [4, 12]. Cephalic and extracephalic cutaneous allodynia induced in rats by activation of dural nociceptors with a cocktail of inflammatory mediators, was also inhibited by oral administration of (S)-LCM [27]. However, the use of this compound is limited by its inhibition of CRMP2-dependent tubulin polymerization [30]. This additional function may result in adverse effects that have not been yet characterized. Another compound that inhibits CRMP2 function in tubulin polymerization is the FDA-approved enantiomer (*R*)-lacosamide (Vimpat) [30]. The fact that a CRMP2 inhibitor is currently used in patients indicates that adverse effects may be negligible or non-existent and reinforces the possibility of targeting CRMP2 to treat chronic neuropathic pain.

## Methods

### Animals

Pathogen-free, adult male Sprague–Dawley rats (250 g; Envigo) were housed in temperature (23 ± 3 °C) and light (12-h light/12-h dark cycle; lights on 07:00–19:00) controlled rooms with standard rodent chow and water available ad libitum. The Institutional Animal Care and Use Committee of the College of Medicine at the University of Arizona approved all experiments. All procedures were conducted in accordance with the Guide for Care and Use of Laboratory Animals published by the National Institutes of Health and the ethical guidelines of the International Association for the Study of Pain. Animals were randomly assigned to treatment or control groups for the behavioral experiments. Animals were initially housed three per cage but individually housed after the intrathecal cannulation on a 12 h light-dark cycle with food and water ad libitum. All behavioral experiments were performed by experimenters who were blinded to the experimental groups and treatments.

### Spared nerve injury (SNI)

Under isoflurane anesthesia (5% induction, 2.0% maintenance in 2 L/min air), skin on the lateral surface of the left hind thigh was incised. The biceps femoris muscle was bluntly dissected to expose the three terminal branches of the sciatic nerve [16]. Briefly, the common peroneal and tibial branches were tightly ligated with 4–0 silk and axotomized 2.0 mm distal to the ligation. Sham animals underwent the same operation; however, the exposed nerves were not ligated. Closure of the incision was made in two layers. The muscle was sutured once with 5–0 absorbable suture; skin was auto clipped. Animals were allowed to recover for 7 days before any testing.

### Immunohistofluorescence and epifluorescence imaging

Tissues were dissected from adult rats and then fixed using 4% paraformaldehyde for 4 h at room temperature (RT). The fixed tissues were transferred into a 30% sucrose solution and left at 4 °C until the sinking of the tissues could be observed (~ 3 days). Tissues were cut at 10 μm thickness using the Bright OTF 5000 Microtome Cryostat (Hacker Instruments and Industries, Inc., Winnsboro, SC), and fixed onto charged slides and kept at − 20 °C until use. Prior to antibody staining, slides were dried at room temperature for 30 min and re-hydrated with phosphate buffered saline (PBS). Next, slices were permeabilized and saturated using PBS containing 3% BSA, 0.3% triton X-100 solution for 30 min at RT, and then primary antibodies (Table 2) were added overnight. The slices were then washed 3X in PBS and incubated with PBS containing 3% BSA and secondary antibodies (Alexa 488 chicken anti-mouse or Alexa 660 chicken anti-rabbit secondary antibodies (Life Technologies)) for at least 3 h at RT. IB4-alexa 594 was added to this step when indicated. After 3 washes (PBS, 10 min, RT), slides were mounted and stored at 4 °C until analysis. Neurotrace (Cat#N21479, Thermo Fisher Scientific) was used to stain neuronal soma. Immunofluorescent micrographs were acquired on a Nikon Eclipse Ti-U (Nikon Instruments Inc.), using objective Super fluor 10 × 0.5 numerical aperture or S Plan Fluor ELWD 20 × 0.45 numerical aperture and a Photometrics Dyno CCD camera (Roper Scientific, Tucson, AZ) controlled by NiS Elements software (version 4.40, Nikon instruments). NiS elements was used to quantify the fluorescence signal on raw images. Briefly, the fluorescence level from individual sensory neuron was quantified and classified based on either the IB4, NF200 or NaV1.8 status of the neurons. The freeware image analysis program Image J (http://rsb.info.nih.gov/ij/) was used to generate the images after a background removal step.

**Table 2:** Antibodies used in this study

Antibody	Species	Catalog number	Company
CRMP2	Rabbit	C2993	Sigma, St. Louis, MO
CRMP2 p522	Rabbit	CP2191	ECM Biosciences, Versailles, KY
NF200	Mouse	MCA1321	AbD Serotec, Raleigh, NC
PGP9.5	Mouse	NB600–1160	Novus Biologicals, Littleton, CO
NaV1.8	Mouse	75–166	UC Davis/NIH NeuroMab Facility
IB4-Alexa 594		I21413	Thermofisher Scientific

### Mechanical Allodynia

Rats were allowed to acclimate within suspended wire mesh cages for 30 min prior to behavioral assessment. Before (pre-baseline), after SNI (post-baseline) and 3, 24, 48, 72 h time points were used to measure response to calibrated von Frey filaments (g) probed perpendicular to the lateral plantar surface of the left hind paw (up-down method). Paw withdrawal thresholds were calculated in grams using the Dixon non-parametric test and expressed as the Paw Withdrawal Threshold (mean ± standard error; SEM) in GraphPad Prism 8.0. All behavior experiments were blinded.

### Statistical analyses

All values represent the mean ± S.E.M. Datasets were analyzed with non-parametric Mann-Whitney and Kruskal Wallis tests (Graphpad Prism 8). A p value of < 0.05 was used to indicate statistical significance between treatment and non-treatment groups.

## Supplementary information

**Additional file 1: Supplementary Fig. 1.** Expression of phosphorylated CRMP2 in the dorsal horn of the spinal cord. Micrographs of a 20 μm section of adult spinal cord from animals having received a spared nerve injury and immunostained with neurotrace to label neuronal cell bodies and an antibody against CRMP2 p522 as indicated. The inset shows an enlarged version of the staining illustrating examples of co-expression of neurotrace with CRMP2 p522.

**Additional file 2: Supplementary Fig. 2.** Expression of CRMP2, Cdk5, neurofibromin, NaV1.7 and CaV2.2 mRNA in DRG subpopulation identified by single cell RNA-seq. Normalized data used to plot the heatmap are also shown (*right*).

## Data Availability

Please contact author for data requests.

## Abbreviations

Cdk5: cyclin dependent kinase 5.
CGRP: calcitonin gene related peptide.
CRMP2: collapsin response mediator protein.
CRISPR: Clustered Regularly Interspaced Short Palindromic Repeats.
DRG: dorsal root ganglia.
IB4: isolectin B4.
NaV1.8: voltage-gated sodium channel subunit 1.8.
SNI: spared nerve injury.
RT: Room temperature.
BSA: bovine serum albumin.
PGP9.5: protein gene product 9.5.
UCHL-1: ubiquitin-C-terminal hydrolase 1.
NF200: neurofilament 200.
TRPV1: transient receptor potential vanilloid 1.
NMDA: N-methyl-D-aspartate.
GSK3β: glycogen synthase kinase-3β.
Lacosamide N-benzyl: 2-acetamido-3-methoxypropionamide.
